# Adrenocortical Carcinoma Steroid Profiles: *In Silico* Pan-Cancer Analysis of TCGA Data Uncovers Immunotherapy Targets for Potential Improved Outcomes

**DOI:** 10.3389/fendo.2021.672319

**Published:** 2021-06-14

**Authors:** João C. D. Muzzi, Jessica M. Magno, Milena A. Cardoso, Juliana de Moura, Mauro A. A. Castro, Bonald C. Figueiredo

**Affiliations:** ^1^ Laboratório de Imunoquímica (LIMQ), Pós-Graduação em Microbiologia, Parasitologia e Patologia, Departamento de Patologia Básica, Universidade Federal do Paraná (UFPR), Curitiba, Brazil; ^2^ Laboratório de Bioinformática e Biologia de Sistemas, Pós-Graduação em Bioinformática, Universidade Federal do Paraná (UFPR), Curitiba, Brazil; ^3^ Instituto de Pesquisa Pelé Pequeno Príncipe, Oncology Division, Curitiba, Brazil; ^4^ Centro de Genética Molecular e Pesquisa do Câncer em Crianças (CEGEMPAC), Molecular Oncology Laboratory, Curitiba, Brazil

**Keywords:** adrenocortical carcinoma, steroidogenesis, PD-1/PD-L1/PD-L2, *PDCD1/CD274/PDCD1LG2*, B7-H3, *CD276*, CD8+ T lymphocytes

## Abstract

Despite progress in understanding the biology of adrenocortical carcinoma (ACC), treatment options have not dramatically changed in the last three decades, nor have we learned how to avoid some of its long-term side effects. Our goal was to improve the understanding of immune pathways that may include druggable targets to enhance immune responses of patients with ACC, focusing on immune evasion and the activation of immune cells against ACC. Our strategy was aimed at improving insight regarding gene expression without steroid interference. Using approaches based on high and low steroid phenotypes (HSP and LSP, respectively), we characterized immune pathways using The Cancer Genome Atlas (TCGA) ACC cohort data. Although previous studies have suggested that patients with ACC receive minimal benefit from immunotherapy, high expression of immune modulators was noted in patients with LSP, suggesting the activation of these biomarkers may be an important adjuvant therapy target after clearance of excess glucocorticoids. In addition, patients with LSP ACC had higher immune cell infiltration than patients with HSP ACC and other cancer subtypes. Our findings can be summarized as follows (1): we confirmed and improved the definition of two immune response pathways to ACC (HSP and LSP) based on *in silico* transcriptome analysis (2), we demonstrated the steroid profile should be considered, otherwise analyses of ACC immune characteristics can generate confounding results (3), among the overexpressed immunotherapy targets, we demonstrated that LSP was rich in *PDCD1LG2* (*PD-L2)* and both HSP and LSP overexpressed *CD276* (*B7-H3)*, which was associated with resistance to anti-PD1 therapy and may have accounted for the modest results of previous clinical trials, and (4) identification of patients with LSP or HSP ACC can be used to help determine whether immunotherapy should be used. In conclusion, we highlighted the differences between LSP and HSP, drawing attention to potential therapeutic targets (*CD276*, *PDCD1*, and *PDCD1LG2*). Treatments to reduce immune evasion, as well as the use of other natural and pharmacological immune activators, should include prior pharmacological inhibition of steroidogenesis. Attempts to combine these with tumor cell proliferation inhibitors, if they do not affect cells of the immune system, may produce interesting results.

## Introduction

Adrenocortical carcinoma (ACC) is a rare highly aggressive malignancy ⁠of which >80% are steroidogenic (1–4)⁠⁠. Overt hypercortisolism is observed in 37.6% (197/524) of adults with ACC, excluding cases with elevated cortisol levels without clinical manifestations, which may also have some molecular impact (4)⁠. The phenotypes seen with molecular changes in ACC may be largely derived from the dominant co-secretion of cortisol and androgens. These differences in combinations of steroid underlie the importance of defining steroid phenotypes and identifying the relevant genetic alterations that characterize the various immune system profiles observed.

Pan-genomic and molecular characterizations of ACC have been the focus of diverse studies in an attempt to establish useful classifications for prognosis or therapy. Zheng et al. (5)⁠ suggested the differentiation of patients with ACC based on three CpG island hypermethylated phenotype (CIMP) statuses: high, intermediate, and low. To date, CIMP is the most robust biomarker for ACC pan-molecular subgroups (6)⁠. Despite providing meaningful prognosis, this classification cannot be translated into determining optimal target treatment options (6, 7)⁠. Besides DNA methylation status, other molecular strategies have been used to characterize ACC subtypes (5, 8, 9)⁠. Based on transcriptome profiling, de Reyniès et al. (8)⁠ and Giordano et al. (9)⁠ defined two ACC subgroups, C1A and C1B, while Zheng et al. (5)⁠ clustered ACC mRNA-seq data in two subgroups with a high steroid phenotype (HSP) and low steroid phenotype (LSP), separating the expression pattern of genes in the steroid synthesis pathway. This was further refined into four subgroups: HSP (n = 25), HSP + proliferation (n = 22), LSP (n = 27), and LSP + proliferation (n = 4). The proliferation profile was mainly associated with HSP, whereas the LSP + proliferation was restricted to only four cases in The Cancer Genome Atlas (TCGA) cohort (n = 78). These four subgroups demonstrated a high overlap of C1A and C1B subtypes, with most HSP patients classified as C1A and LSP patients classified as C1B (5)⁠. Zheng and colleagues found an immune signature associated with the LSP profile upon comparison of immune-suppressed ACC with strong steroidogenesis (5)⁠.

Landmark studies have extensively focused on profiling biomarkers and cells associated with cancer immunity with extensive immunogenomic analysis of tumors (10, 11)⁠. Thorsson et al. (11)⁠ provided new insights into the immune landscape of cancers using TCGA data. Assembling all ACC cases without consideration of more specific clinical or steroidal phenotypes, ACC was found to exhibit the second lowest leukocyte fraction among the cancer types studied (11). These investigators analyzed over 10,000 TCGA tumors and using cluster analysis of a robust list of immune expression signatures reported by other studies proposed six immune subtypes with specific features and prognoses: C1, wound healing; C2, IFN-γ dominant; C3, inflammatory; C4, lymphocyte depleted; C5, immunologically quiet; and C6, TGF-ß dominant (11). Based on the dominant sample characteristics, they proposed the ACCs in the TCGA database (patient ages ranging from 14 to 77 years) fit better within the C4 immune subtype, with a more prominent macrophage signature, suppression of T helper type 1 (Th1) cells, and a high M2 response (11). These cancers are among those with the least favorable outcome. This immunosuppressed profile is associated with hypercortisolism and low survival in the majority of adult patients with ACC (12)⁠, while younger children with a dominant androgen steroid phenotype have a better prognosis (13, 14)⁠.

As tumors grow, they acquire mutations. These mutations may impair the function of the immune system in ACC subtypes (with both low and high steroidogenesis). These genetic changes include mutations in driver genes, DNA damage, copy number variation, aneuploidy, loss of heterozygosity, intratumor heterogeneity, and decreased telomere length (5, 15)⁠. Consequently, overall survival (OS) analyses are frequently less accurate in ACC cases because of the complex interactions between these changes in the tumor and the immune system (16)⁠. The worse prognosis for adults with ACC is explained by molecular differences and the higher frequency of elevated glucocorticoids (17–19)⁠. After C4 (49 of the 78 ACC cases), the second most common immune subtype was C3, found in 23 of the 78 ACC cases, predominantly in LSP (16/31). The C3 immune subtype is represented by an inflammatory response with higher presence of Th1 and Th17 responses and a balanced presence of macrophages and lymphocytes. In a pan-cancer analysis, the C3 immune subtype presented the best prognosis. The other six ACC cases were C5 (n = 3) and one each of subtypes C1, C2, and C6.

Lymphocyte expression signature, high number of unique T-cell receptor clonotypes, cytokines produced by activated CD4+ Th1 and Th17 lymphocytes, and M1 macrophages, which secrete pro-inflammatory cytokines and chemokines, are associated with improved OS in some cancer subtypes (11). The ability to kill transformed cells is essential for an effective anti-tumor response by the immune system. This ability relies on the cytotoxic activity of natural killer (NK) cells and CD8+ T lymphocytes (CD8+TL) (20)⁠. Activated NK cells are able to recruit dendritic cells and elicit an antitumor T-cell response (21)⁠. The presence of activated tumor-infiltrating CD8+TL in human cancers is associated with improved prognosis, expression of specific antigens, and increased immune-cancer interactions (10)⁠. For example, there is a positive correlation between melanoma-associated antigen 3 (*MAGEA3*) expression and CD8+TL infiltration in melanoma, explaining the success of the *MAGEA3*-based vaccine (22)⁠. Another successful example of immunomodulation is the stimulation of blood cells with bacille Calmette-Guérin (BCG) or recombinant BCG to enhance the expression of CD25 and CD69 on human CD4+ T cells, a highly effective immunotherapeutic agent against bladder cancer (23, 24)⁠. Interestingly, most patients with bladder cancer are diagnosed in their seventies when the immune system may be impaired (25)⁠. Similarly, more than 50% of adults with ACC are diagnosed in patients 50 years or older (26)⁠. As CD8+TL are crucial for protective immunity, it is vital to identify candidate activators of the host immune response to cancer. Specific driver mutations (e.g., *TP53*) are correlated with higher leukocyte levels across all cancers (11). This is not the case in pediatric ACC with the germline *TP53* R337H variant, where higher CD8+TL infiltration is associated with other factors, such as stage 1 and diagnosis at a younger age (14)⁠. This is in line with the observed changes in the immune infiltrate composition at each tumor stage, where there is a decrease in T-cell density and an increase in T-follicular helper cells and innate immune cells with tumor progression (27)⁠.

T-cell activation requires two distinct signaling pathways, one mediated by the binding of T-cell receptor (TCR) with an antigen bound major histocompatibility complex (MHC) and the second by the binding of CD28 to T cells with B7-1 (CD80) or B7-2 (CD86) of antigen-presenting cells (28)⁠. In addition to B7-1 and B7-2, other B7 family proteins interact with other T-cell membrane molecules and may act as co-inhibitors, such as B7-H1, which is well known as programmed cell death ligand 1 (PD-L1), B7-DC, which is known as programmed cell death ligand 2 (PD-L2), and B7-H3 (CD276), among others (29)⁠. Blockade of immune checkpoint evasion was expected to provide a more effective treatment for patients with ACC. However, the use of antibodies targeting programmed cell death 1 (PD-1) expressed by T cells (30–32)⁠ or PD-L1 (33)⁠ is associated with poor efficacy. In the latter study (33)⁠, 50% of patients were under concomitant treatment with mitotane to block immunosuppressive glucocorticoids. However, this study did not consider the possibility of evasion through PD-1/PD-L2. Despite these setbacks, other approaches have been suggested to overcome the reported modest responses (7, 34)⁠.

The impact of excess steroids on the immune system interaction with ACC tumors, including a complete understanding of the related molecular profiles, has not been fully evaluated. Our aim was to perform a comprehensive and translational analysis of ACC immune biomarkers to identify those that could predict a favorable outcome with and without steroid interference. We evaluated possible modifiers of immune evasion (*CD274* that encodes PD-L1 and *PDCD1LG2* that encodes PD-L2), and investigated how these ligands correlate with *CD8B* expression in ACC, revisiting TCGA cohort data and the molecular steroidogenic classification established by Zheng et al. (5). Considering the hypothesis that HSP has a worse prognosis because of its effect on the immune system, we discuss strategies, supported by prior studies, for downregulating steroid biosynthesis before using possible immune activators.

## Materials and Methods

### Data Sources

Data sources used in this study were obtained from the ACC cohort in TCGA (5). Inclusion criteria, quality control, and characterization of these participants were previously described (5). The data are available on the National Cancer Institute (NCI) Genomic Data Commons (GDC) Data Portal (https://gdc.cancer.gov/about-data/publications/acc_2016). The transcriptome profiles of 78 patients with ACC for whom the steroid phenotype was available were used for the subsequent analysis. This group included 31 men and 47 women with a mean age of 46.7 years (range, 14–77 years). Based on mRNA data, the patients were classified as HSP (n = 25), HSP + Proliferation (n = 22), LSP (n = 27), and LSP + Proliferation (n = 4) (5). For the current study, we used only the steroid phenotype classification, HSP (n = 47) and LSP (n = 31). Clinical data were obtained using the R/Bioconductor package TCGABiolinks v 2.18.0 (35–37)⁠ from the GDC Data Portal. History of excessive hormone expression was downloaded from the GDC Data Portal and classified as (i) Cortisol Excess (n = 32) for patients with a history of excess Cortisol (n = 15), Mineralocorticoids|Cortisol (n = 1), and Androgen|Cortisol (n = 16); (ii) Mineralocorticoids Excess (n = 4) for patients with a history of Mineralocorticoids (n = 3) and Mineralocorticoids|Cortisol (n = 1); and (iii) Sexual Hormone Excess (n = 28) for patients with a history of excess Androgen (n = 8), Androgen|Cortisol (n = 16), Androgen|Estrogen (n = 2), and Estrogen (n = 2). There were no notations on hormone levels for 5 patients.

The data on immune characteristics were obtained from Thorsson et al. (11) (https://www.cell.com/cms/10.1016/j.immuni.2018.03.023/attachment/1b63d4bc-af31-4a23-99bb-7ca23c7b4e0a/mmc2) and xCell immune infiltrate cell scores were downloaded from TIMER2.0 (http://timer.cistrome.org/infiltration_estimation_for_tcga.csv.gz) (10, 38, 39)⁠. For comparison between two steroid groups, data were filtered using the patients’ barcodes.

The study was approved by the Ethics Committee of Pequeno Príncipe Hospital and National Research Ethics Committee (CAAE number 50622315.0.0000.0097), without use of a consent form as it involved only anonymous public data from the original cohorts deposited at TCGA.

### Leukocyte Fraction and Immune Infiltrate Comparison

Leukocyte fractions data for pan-cancer analysis were extracted from Thorsson et al. (11). The study included 11,080 patients from TCGA of which 10,448 were annotated with a leukocyte fraction. TCGA studies were used and were arranged by median leukocyte fraction values. For grouping based on the ACC steroid phenotype, patients from this cohort were selected according to their barcode identifiers.

Previous studies used two methods to estimate immune cell infiltration in the ACC cohort: xCell (38)⁠ scores using a marker-gene-based approach, inferred by Sturm et al. (39)⁠, and Cibersort using a deconvolution-based approach (40)⁠, calculated by Thorsson et al. (11). A more detailed evaluation on the limitations and characteristics of these approaches has been reviewed by Sturm et al. (39)⁠.

The immune cell proportions based on Cibersort estimations were multiplied by the leukocyte fraction, resulting in a final score that could be used for intra- and inter-sample comparisons, as previously described (11). For the ACC cohort analysis, scores of CD8+TL and activated NK cells were used for pan-cancer comparisons, while the scores for all 22 cell types were used for intra-cohort analysis. For pan-cancer comparisons, the barcodes were merged with the Thorsson dataset, and xCell results for CD8+TL and NK cells of the TCGA study and were grouped and ordered by median values. For comparisons within the ACC cohort, samples were filtered for all xCell infiltrate estimates by patient barcodes. Beyond cell estimations, xCell offers microenvironment, immune, and stroma scores. However, the enrichment scores do not allow for a comparison between different cell types of the same sample, but does allow for comparison of the same cell type between different samples. Accordingly, the two groups of patients could be compared using this method, but the relative abundance of each cell type within a patient could not be compared.

The Wilcoxon test was used to compare the xCell and Cibersort immune infiltrate scores between the HSP and LSP groups. The R package ComplexHeatmap v 2.6.2 (41)⁠ was used for heatmap construction. Column-wise z-scores were calculated for the immune cell scores, and maximum and minimum values were limited to +2 and −2, respectively. Samples were clustered for immune characteristics and cell scores scaled data in a semi-supervised manner using the McQuitty and Euclidean distance methods.

### RNA-seq Filtering, Processing, and Differential Expression (DE)

High-throughput sequencing (HTSeq) mRNA data were obtained for the 78 ACC patients with steroid phenotype characterization using the R package TCGAbiolinks v 2.18.0 (35–37)⁠ and SummarizedExperiment v 1.20.0 (42)⁠. The 56,457 transcripts were filtered for protein-coding genes using EnsembleDb version 101 and notated using AnnotationHub R package v 2.22.0 (43)⁠. The genes with a sum of zero counts were excluded, resulting in the inclusion of 19,169 genes. The two steroid phenotype groups underwent DE analysis using the R package DESeq2 v 1.30.0 as previously described (44)⁠. P-values were calculated using Wald’s test and adjusted with Benjamini–Hochberg estimation. The gene count expression matrix was normalized by variance-stabilizing transformation using the DESeq2 package. Three genes had duplicated symbol names (*PINX1, TMSB15B*, and *MATR3*). To differentiate these three genes, we added an underscore after the first entry in the DE results.

### Enrichment Analysis

After ordering based on adjusted p-values, the ranked genes list underwent gene set enrichment analysis (GSEA) with the Coincident Extreme Ranks in Numerical Observations (CERNO) test (45, 46)⁠ using the R package tmod v 0.46.2, as previously described (46, 47)⁠. The CERNO test is an application of modified Fisher p-value integration for creating a GSEA ranked list, which avoids the arbitrary choice of a p-value or log-fold change threshold (46)⁠. Two gene sets were used for the enrichment analysis. Hallmark gene sets were obtained from MSigDb v7.1 (https://data.broadinstitute.org/gsea-msigdb/msigdb/release/7.1/msigdb_v7.1.xml) (48, 49)⁠ and blood transcriptional modules (BTM) (50)⁠ were obtained from the tmod R package. The hallmark gene set is comprised of 50 modules summarizing the molecular signature database gene sets and reducing redundancy (49)⁠. These modules are divided into eight categories: cellular component, development, DNA damage, immune, metabolic, pathway, proliferation, and signaling. The other gene set consists of 334 BTMs combining information about adaptive and innate immune responses.

### Expression of Immune Modulators and Immune Checkpoints

Expression profiles of the immunomodulatory genes listed by Thorsson et al. (11) (https://www.cell.com/cms/10.1016/j.immuni.2018.03.023/attachment/8d3ffc74-4db4-4531-a4ad-389dfc8bb7ec/mmc7.xlsx) were obtained from a variance stabilizing transformation gene expression matrix. Of the 75 immune modulators, only one (*C10orf54*) was not found in the expression matrix. *CSF2* expression was added based on the study by Wang et al. (51)⁠, which showed its importance for dendritic cell recruitment in poorly immunogenic tumors. Adjusted p-values for the findings from the DE analysis were used for comparison between groups. The R package ComplexHeatmap v 2.6.2 (41)⁠ was used for heatmap construction, column-wise z-scores were calculated for immune modulator gene expressions, and maximum and minimum values were limited to +2 and −2, respectively. Columns were arranged following the order of the semi-supervised clusters of immune characteristics and cell scores determined previously. For expression of immune checkpoint genes *CD8B*, *CD274* (*PD-L1)*, and *PDCD1LG2* (*PD-L2)*, Pearson’s correlation coefficients and p-values were calculated and the confidence interval was inferred based on Fisher’s Z transformation.

### Leukocyte Fractions and Mutations in the ACC Cohort

Pan-Cancer Atlas data from TCGA regarding ACC patients with mutations in *TP53* (n = 16), *CTNNB1* (n = 13), and *MEN1* (n = 7) were obtained from the cBioPortal (https://www.cbioportal.org/) and merged with the leukocyte fraction information. Comparison between patients with and without mutations in these driver genes was performed using the Wilcoxon test.

### Extracellular Communication Networks in Immune Subtypes

Extracellular communication networks were inferred based on the study by Thorsson et al. (11) and the data for the TCGA ACC cohort were obtained from iAtlas (https://isb-cgc.shinyapps.io/shiny-iatlas/). The networks involved ligand-receptor, cell-receptor, and cell-ligand pairs described by Ramilowski et al. (52)⁠ and enhanced by Thorsson et al. (11). Nodes with more than 50% abundance and concordance interaction greater than 2.5 were chosen as the C3 and C4 immune subtypes. The networks were reconstructed using R packages igraph v 1.2.6 and RedeR v 1.38.0 (53)⁠. The node line width varies proportionally to the abundance score and the edge width varies proportionally to the concordance.

A batch corrected and normalized gene expression matrix for the 11,060 TCGA samples was downloaded from the Supplemental Data of Hoadley et al. (54)⁠ (https://api.gdc.cancer.gov/data/9a4679c3-855d-4055-8be9-3577ce10f66e). The matrix was filtered for the 25 ligands and receptors present in the C3 and C4 networks. Samples were selected from primary tumors only and duplicate entries were removed. The matrix was merged with the Thorsson dataset according to the participant barcode. Gene expression data were ranked from lowest to highest counts for each gene, and patients were divided into terciles (low, intermediate, and high) and filtered for the ACC patients’ barcodes.

### Survival Analysis

The prognostic impact of LSP (n = 31) versus HSP (n = 47), immune subtype C3 (n = 23) versus C4 (n = 49), and LSP + Immune Subtype C3 (n = 16) versus other configurations (n = 62) were evaluated using Kaplan-Meier analysis for OS and progression-free interval (PFI) using R packages survival v3.2-7 (55, 56)⁠ and survminer v0.4.8. P-values were calculated using the log-rank test.

### Statistical Analysis

Q values and false discovery rate (FDR) quantities were calculated using R package qvalue v2.20.0 (57)⁠. For DE results, q-values were calculated based on π estimation. Due to the small number of p-values available for π estimation, the q-value was determined using Benjamini-Hochberg estimation for enrichment analysis.

## Results

### Pan-Cancer Comparison Showed Significant Immune Infiltrates in Patients With LSP ACC

**Figure 1:**
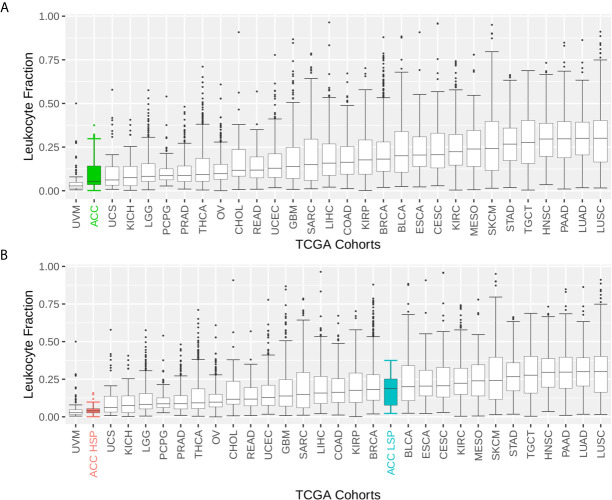
Boxplot of the leukocyte fraction in the tumor microenvironment of 30 TCGA solid tumors. The leukocyte fraction was inferred by Thorsson et al. (11) for 10,448 participants included in The Cancer Genome Atlas (TCGA) database using molecular methods and compared with hematoxylin and eosin stained images, which demonstrated good correlation. Participants were grouped by TCGA cohort. The boxplot shows the median and interquartile range (IQR) for the leukocyte fraction of each cohort. Whiskers are set at 1.5 IQR and outliers (above and below the 1.5 IQR) are presented as points. TCGA cohorts were ordered by median values. **(A)** Patients with adrenocortical carcinoma (ACC) are represented as one group. **(B)** Patients with ACC are divided according to steroid phenotypes.

**Figure 2:**
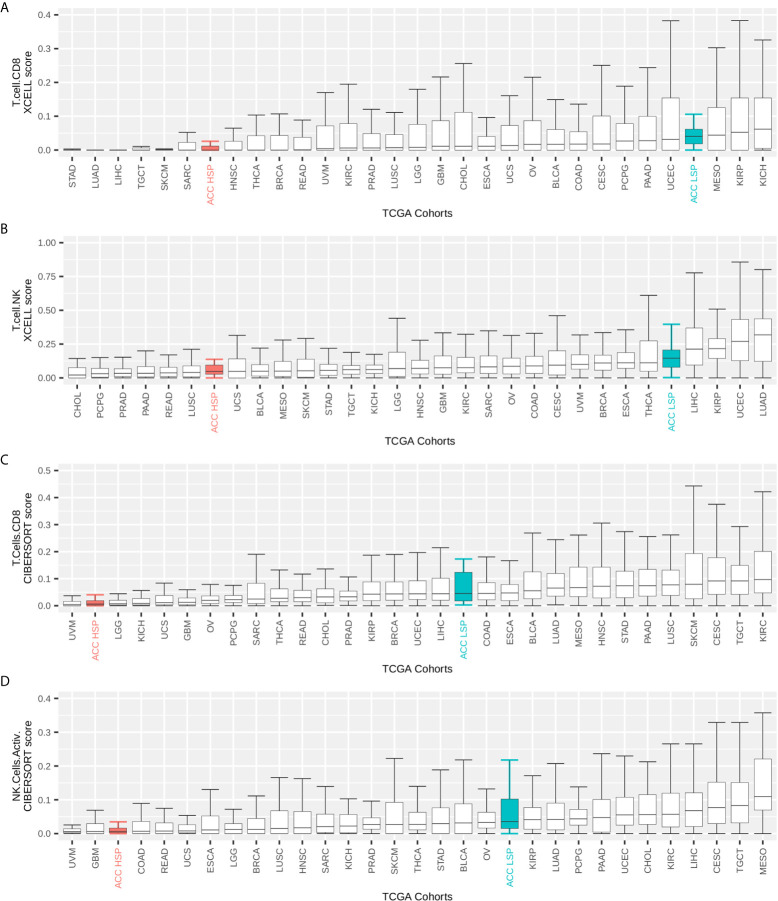
TCGA pan-cancer comparison of immune infiltrates. Immune infiltrate scores (y-axis) were rescaled to range from 0 to 1. The maximum on the y-axis is independently set for each plot for better visualization. Participants were grouped by TCGA cohort and the boxplot shows median and IQR for immune cell infiltrate scores of each cohort. Whiskers are set at 1.5 IQR and outliers (above and below the 1.5 IQR) are not shown. TCGA cohorts were ordered by median values. Patients with ACC were divided according to steroid phenotypes **(A, B)** xCell scores for CD8+ T cells and T natural killer (NK) cells inferred by Sturm et al. (39). **(C, D)** Cibersort scores for activated CD8+ T cells and NK cells as inferred by Thorsson et al. (11) and multiplied by the leukocyte fraction as described by these authors.

ACC had one of the lowest immune infiltrate profiles in the TCGA pan-cancer comparison (**Figure 1A**). However, when distinguishing by steroid profiles, patients with LSP ACC had considerable leukocyte infiltration, placing them in the top 40% of tumor types (**Figure 1B**). This was also seen with CD8+TL and NK cells infiltrates, as measured by the Cibersort and xCell scores (**Figures 2A–D**).

The main clinical characteristics of HSP and LSP are shown in **Table 1**. Cortisol was the most frequently elevated hormone (57.4% in HSP and 16.1% in LSP) compared to less frequently observed sex steroids, mineralocorticoids, and others, as described by Zheng et al. (5). The prognostic differences were remarkable, with only 3 death events (9.7%) in patients with LSP versus 24 (51.1%) in patients with HSP. This was in addition to a higher proportion of advanced cancer stages observed in the HSP group, which may have been associated with a higher proliferation rate.

**Table 1:** Clinical data.

	HSP	LSP
**Total (Dead)**	47 (24)	31 (3)
**Mean Age ± SD**	46.5 ± 17	48.5 ± 14.3
**Males Mean age ± SD**	16 (9)	15 (2)
	46 ± 14.6	52.9 ± 11
**Females Mean age ± SD**	31 (15)	16 (1)
	46.8 ± 18.3	44.3 ± 16.1
**High Proliferation**	22 (15)	4 (2)
**Tumor Stage**		
**Stage I**	3 (1)	6 (0)
**Stage II**	18 (6)	19 (1)
**Stage III**	12 (6)	4 (1)
**Stage IV**	12 (10)	2 (1)
**Hormone Excess**		
**Cortisol**	27 (14)	5 (1)
**Mineralocorticoids**	3 (1)	1 (0)
**Sexual**	20 (14)	8 (1)
**Immune Subtype**		
**C1**	1 (1)	0 (0)
**C2**	0 (0)	1 (1)
**C3**	7 (3)	16 (0)
**C4**	37 (19)	12 (1)
**C5**	2 (1)	1 (0)
**C6**	0 (0)	1 (1)

The total number of patients for each clinical category for the Low Steroid Phenotype (LSP) and High Steroid Phenotype (HSP) is shown with the number of dead patients in parenthesis. The mean age of participants with the standard deviation (SD) is shown for both groups and subdivided into males and females. All clinical data were retrieved from Zheng et al. (5) and the immune subtype classification from Thorsson et al. (11). Proliferation is bipartite as presented by Zheng et al. (5) based on transcriptomic classification and associated with markers such KI67 and BUB1.

### Immune Activation Was Enriched in Patients With LSP ACC

**Figure 3:**
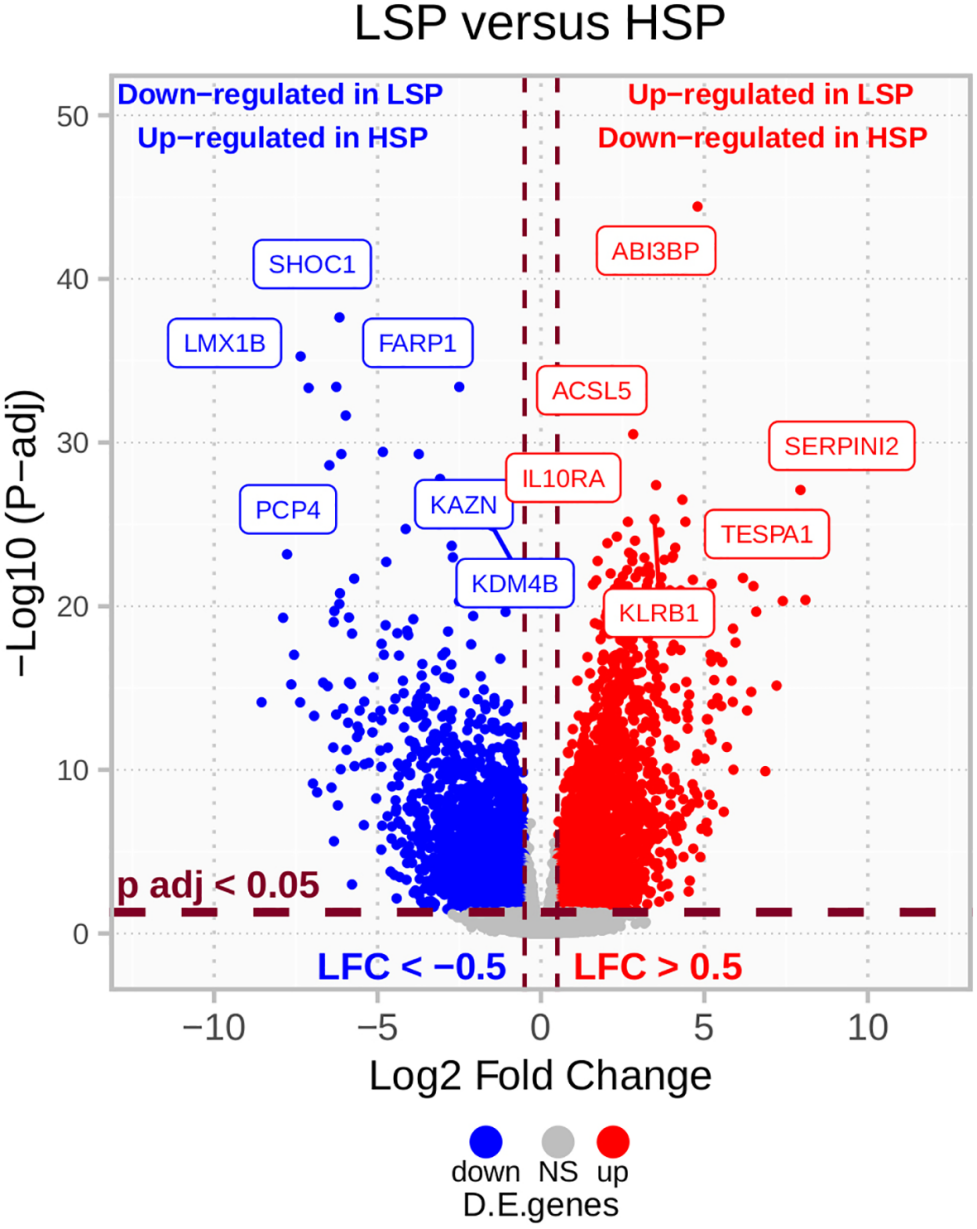
LSP *versus* HSP DE in TCGA ACC cohort (n = 78). After filtering for protein-coding genes and excluding those with sums of zero counts, a total of 19,169 genes were used for a LSP *versus* HSP differential expression (DE) analysis. P-values were calculated using the Wald’s test and adjusted using Benjamini-Hochberg estimation. Genes with absolute log2 fold change lower than 0.5 and adjusted p-value greater than 0.05 are colored in gray. Above these cut-offs, genes upregulated in LSP and downregulated in HSP are indicated in red and genes downregulated in LSP and upregulated in HSP are indicated in blue.

The results from the DE analysis of LSP versus HSP are presented in **Figure 3**. The order is based on LSP versus HSP and is used in all analyses presented in this study. Opposite values for the log-fold change would have been determined if HSP versus LSP was used. The full DE results are available in **Supplementary Table 1**.

**Figure 4:**
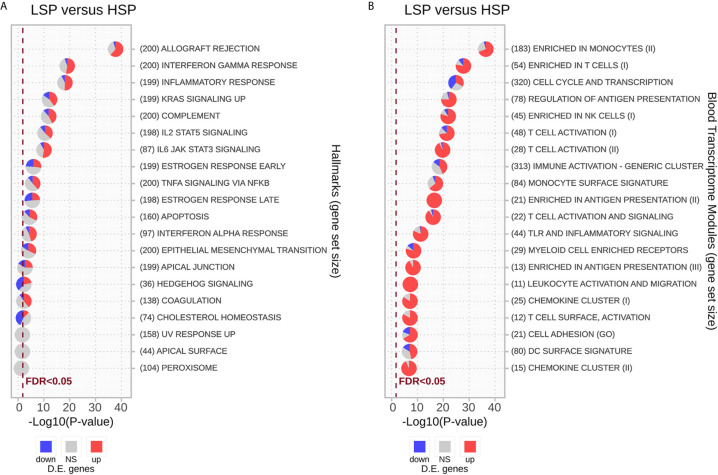
Enrichment analysis for differentially expressed genes using the CERNO test. The CERNO test was applied to the differentially expressed genes using the hallmark gene sets **(A)** and blood transcriptome modules (BTMs) **(B)**. The modules are ordered according to the adjusted p-values and are considered non-significant based on a false discovery rate (FDR) of 0.05. The number of genes in each module is presented before the module names in brackets. The charts are colored based on the DE results: red for module genes upregulated in low steroid phenotype (LSP) and downregulated in high steroid phenotype (HSP), gray for module genes with no significant p-value, and blue for module genes down-regulated in LSP and upregulated in HSP.

Enrichment analysis using the hallmark gene sets is presented in **Figure 4A** and **Supplementary Table 2**. Most gene sets were enriched for genes related mainly to the immune response and were upregulated in LSP and downregulated in HSP. The gene sets significantly enriched with genes downregulated in LSP and upregulated in HSP were associated with estrogen response, cholesterol homeostasis, and hedgehog signaling (**Figure 4A**), which was consistent with the steroidogenic profile used for the DE analysis.

As the immune response was strongly associated with the different steroid phenotypes, we conducted GSEA with the BTMs described by Li et al. (50)⁠. In general, modules associated with T lymphocytes and NK cells, monocytes, and antigen presentation displayed the strongest association with ranked genes, especially those upregulated in LSP. With exception of the “Cell Cycle and Transcription” module, most immune modules were markedly enriched with genes up-regulated in LSP and down-regulated in HSP, with some modules such as “Enriched in Antigen Presentation (II)” and “Leukocyte Activation and Migration” fully represented by DE genes up-regulated in LSP (**Figure 4B**).

### Enrichment of T Cells, Monocytes, and Cytolytic Activity in LSP ACC

**Figure 5:**
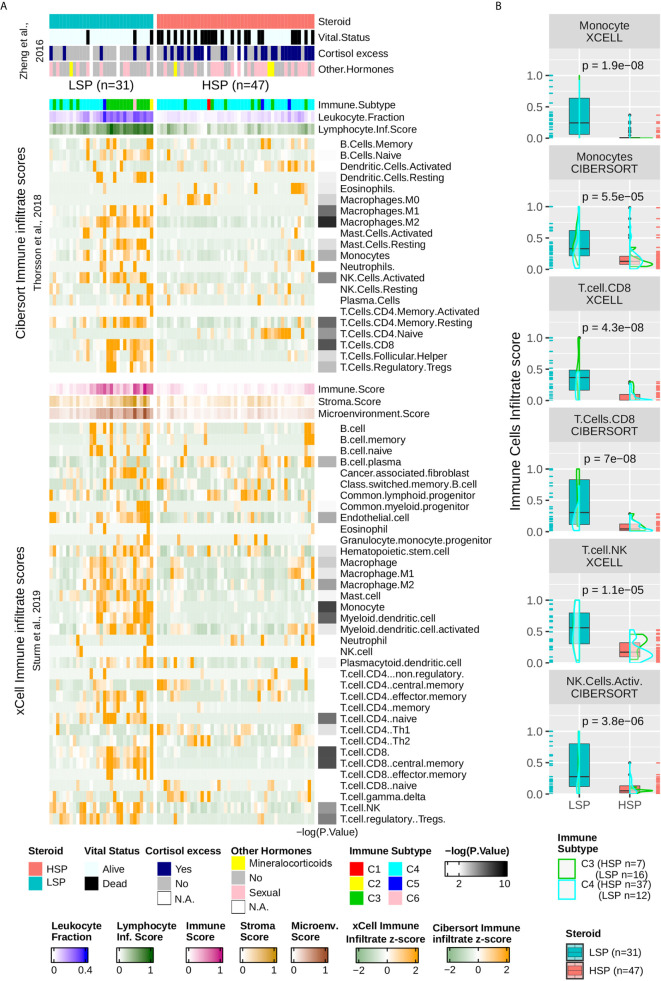
Immune infiltrate comparison. **(A)** Heatmap with column-wise z-scores for TCGA ACC immune infiltrates as inferred by Sturm et al. (39) using xCell and by Thorsson et al. (11) using Cibersort. Maximum and minimum z-scores for the color gradient were set at +2 and −2 standard deviations from the mean. Each column represents a patient and columns were subjected to semi-supervised clustering. In addition to the immune infiltrate, Thorsson et al. (11) presented a classification of six immune subtypes (C1–C6) and inferred the leukocyte fraction and a molecular signature associated with the lymphocyte infiltrate score. xCell provides a stroma, immune, and microenvironment score in addition to the immune cell infiltrate. Lymphocyte infiltrate, immune, stroma, and microenvironment scores were rescaled to a range of 0 to 1. The clinical information was provided by Zheng et al. (5) and appears in the top annotation rows. The p-value calculated using the Wilcoxon test comparing LSP and HSP for each cell type is represented in the right as a color gradient ranging from 2 to 10 for −log10 (p-value), values lesser than 2 are set as white. **(B)** Boxplot comparing LSP and HSP in terms of monocytes, CD8+ T cells (xCell and Cibersort), T natural killer (NK) cells (xCell), and activated NK cells (Cibersort). All scores (y-axis) are rescaled from 0 to 1. The density plot shows the distribution according to the immune subtypes: C3 (green) and C4 (cyan). Each sample is shown in a rug plot and colored according to the HSP and LSP classification. The p-value calculated using the Wilcoxon test comparing both phenotypes is shown above each pair of boxplots.

The column-wise z-scores for the xCell and Cibersort results for TCGA ACC patients are presented in **Figure 5A**. Both methods found a higher immune cell presence in patients with LSP compared to those in patients with HSP, which appeared immunosuppressed. Not all cell types identified using one method were present in the results obtained using the other method, indicating differences in some specificities. Consistent with the BTM enrichment analysis (**Figure 4B**), the presence of monocytes and T cells differed significantly between the two steroid phenotypes (**Figures 5A, B**). As xCell detected NK cells in only a few samples and Cibersort differentiated resting NK cells from activated NK cells but did not distinguish between subtypes of CD8+ T cells, the last two panels of **Figure 5B** show a boxplot comparison of the xCell T NK cells and the Cibersort activated NK cells. Because xCell generates arbitrary scores, y values were rescaled to a range of 0 to 1. Most discrepancies between the xCell and Cibersort scores were observed for B cells, plasma cells, naive CD4+ T cells, and eosinophils, which may have been a result of the spillover effect from the methods (39).

### LSP Was Associated With an Inflammatory Immune Response and HSP With a Lymphocyte Depleted Microenvironment

According to the classification by Thorsson et al. (11), ACC patients fit mainly into the C4 subtype (n = 46), with a subset of patients in C3 subtype (n = 23), where C3 seemed to be associated with LSP (n = 16) and C4 with HSP (n = 37) (**Table 1** and **Figure 5A**). Only one participant each fit into the C1, C2, and C6 subtypes, and three patients presented with a C5 subtype profile. Density plots show the distribution of C3 and C4 ACC patients separated into the steroid phenotypes based on monocyte, CD8+TL, and NK cell scores (**Figure 5B**).

**Figure 6:**
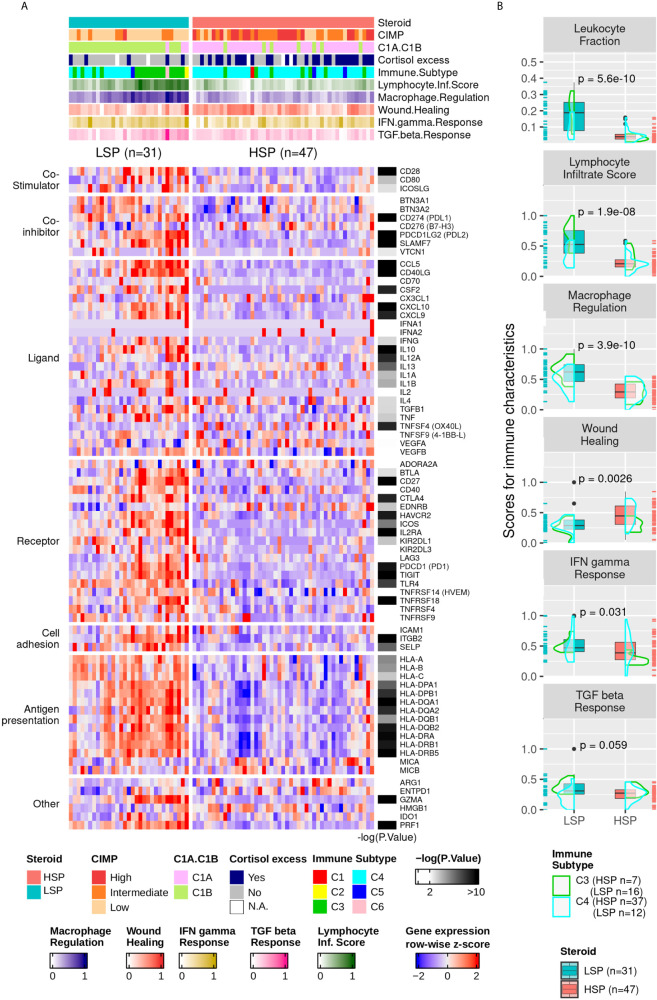
Gene expression of immune modulators and Thorsson and colleagues’ immune signatures. **(A)** Heatmap with column-wise z-scores for expression of immune modulator genes. The maximum and minimum z-scores for the color gradient is set at +2 and −2 standard deviations from the mean. Each column represents a patient. The order from **Figure 5A** was maintained. The p-value for the DE analysis for each gene determined using the Wald’s test is represented in the right. The −log10 (p-value) color gradient is set as white up to the minimum value of 2 and the maximum is set to 10; however, its range exceeds 20 for *GZMA, CCL5*, and *CD40LG*. The absolute p-values for all differentially expressed genes can be found in **Supplementary Table 1**. *Clinical cortisol excess and the molecular classification were described by Zheng et al. (5) and presented in the top annotation. **Immune subtype classification and the five molecular immune signatures rescaled to a range of 0 to 1 were according to Thorsson et al. (11) and are shown in the top annotation. **(B)** Boxplot comparing LSP and HSP for leukocyte fractions and the five immune signatures associated with lymphocyte infiltrate scores, macrophage regulation, wound healing, IFN-γ response, and TGF-ß response according to Thorsson et al. (11). The scores for each of the five signatures are rescaled to a range of 0 to 1 (y-axis). The density plot shows the distribution according to the immune subtypes C3 (green) and C4 (cyan). Each sample is shown in a rug plot and colored according to HSP and LSP classification. The p-value calculated using Wilcoxon test comparing both phenotypes is shown above each pair of boxplots.

The higher immune activation of patients with LSP ACC was also seen in the expression of immunomodulator genes (**Figure 6A**). Patients with LSP had a higher expression pattern of immune activators and inhibitors compared to patients with HSP. From the immune characteristics analyzed by Thorsson et al. (11), the differences between LSP and HSP were significant for leukocyte fraction, lymphocyte infiltration score, and macrophage regulation (**Figure 6B**), which demonstrates immune activation and recruitment in patients with LSP, in contrast with the immunosuppressed tumor microenvironment (TME) seen in patients with HSP.

Thorsson et al. (11) also evaluated the T-cell receptor (TCR) and B-cell receptor (BCR) repertoire. However, this information for most patients with ACC unfortunately could not be retrieved from bulk mRNA sequencing, probably due to the small numbers of these cell fractions in the TME. For instance, only 5 of 78 patients had information regarding BCR (3 LSP and 2 HSP) and 37 regarding TCR (21 LSP and 16 HSP) of which 16 had a 0 score on TCR Shannon entropy (3 LSP and 13 HSP).

### Steroid Phenotype Generated a Confounding Effect on the Correlation Between Leukocyte Fraction and Driver Gene Mutations

Thorsson et al. (11) reported that mutations in driver genes are significantly correlated with the leukocyte fraction. Among driver genes, the most common mutations found in the ACC cohort were in *TP53* (n = 16), *CTNNB1* (n = 13), and *MEN1* (n = 7); however, these mutations were mainly present in patients with HSP (n = 13 of 16, n = 13 of 13, and n = 6 of 7, respectively). It was not possible to determine whether differences in the leukocyte fraction were caused by the mutations or due to the steroid phenotype. Using the Wilcoxon test to evaluate the differences in the leukocyte fraction based on the presence or absence of these mutations resulted in p-values of 1.8 × 10^-1^, 3.8 × 10^-2^, and 4.8 × 10^-1^ for *TP53*, *CTNNB1*, and *MEN1*, respectively.

### Extracellular Communication Networks and Possible Immunotherapy Targets Associated With Steroid Phenotypes

**Figure 7:**
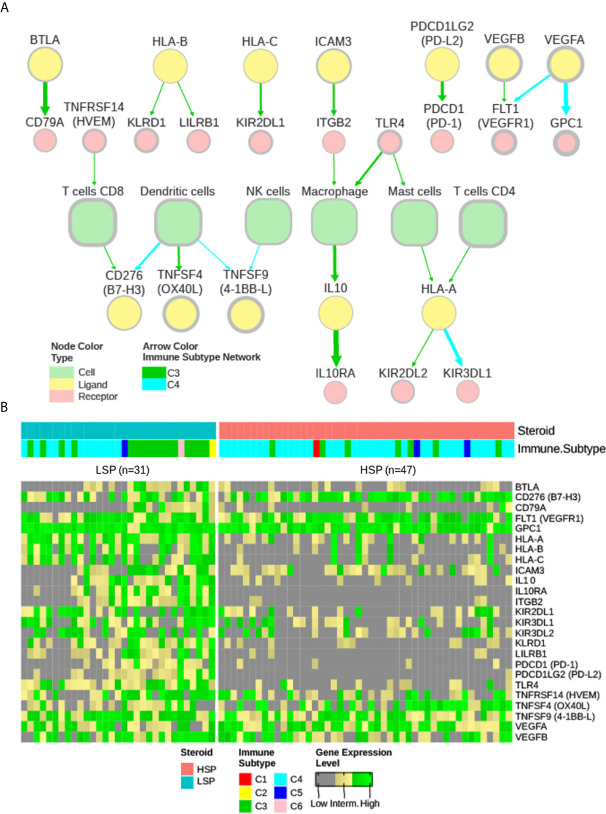
iAtlas explorer-extracellular communication network. **(A)** The extracellular communication network according to Thorsson et al. (11) and available on CRI Atlas (https://isb-cgc.shinyapps.io/shiny-iatlas/) was reconstructed for the C3 and C4 patients of TCGA ACC cohort. Nodes with greater than 50% abundance and a concordance interaction larger than 2.5 were selected. The node line width varies proportionally to abundance and the edge width varies proportionally to concordance. Green arrows represent interactions of C3 patients and cyan arrows represent interactions of C4 patients. Node colors represent immune cells (light green), receptors (pink), and ligands (yellow). **(B)** The receptors and ligands present in the network were evaluated based on gene expression levels in a pan-cancer comparison. Gene expression of the 25 nodes in the network were ranked among 9,361 primary tumor samples available in the TCGA database and classified as low (grey), intermediate (yellow), or high (green) expression level based on ranking terciles. Each column represents a patient with ACC. Column order is maintained from **Figures 5A** and **6A** heatmaps. Steroid phenotype and immune subtype classifications are indicated in the top annotation.

Based on the study by Thorsson et al. (11), two extracellular communication networks were reconstructed for ACC subtypes C3 and C4 with 50% abundance and 2.5 concordance thresholds (**Figure 7A**). A small network was also obtained for the C5 subtype with these parameters centered on macrophages; however, as it contained only 3 patients and was not further considered in the current study. The abundance was representative of the prevalence of the node in the samples (%), while concordance was relative to the concordance to discordance ratio (a pair with concordant high or low expression versus a pair with distinct expression patterns in each sample).

The nodes in the C3 and C4 networks were used to compare all TCGA participants. The ACC patient classification is shown in **Figure 7B**. Most ACC patients, despite phenotype distinction, were in the top third group for expression of *CD276 (B7-H3)*, *GPC1*, vascular endothelial growth factor A (*VEGFA)*, vascular endothelial growth factor B (*VEGFB)*, and vascular endothelial growth factor receptor 1 *(VEGFR1) FLT1* (**Figure 7B**).

Despite its greater representativity in the ACC cohort (49 of 78 patients), C4 formed a smaller network (**Figure 7A**) with generally lower abundance and weaker connections. Curiously, a high abundance of *TNFSF9* (*4-1BB-L)*, commonly expressed by antigen-presenting cells, was found to be related to the C4 network, but not its receptor *TNFRSF9* (*4-1BB)*, which is normally expressed in CD8+ T cells.

**Figure 8:**
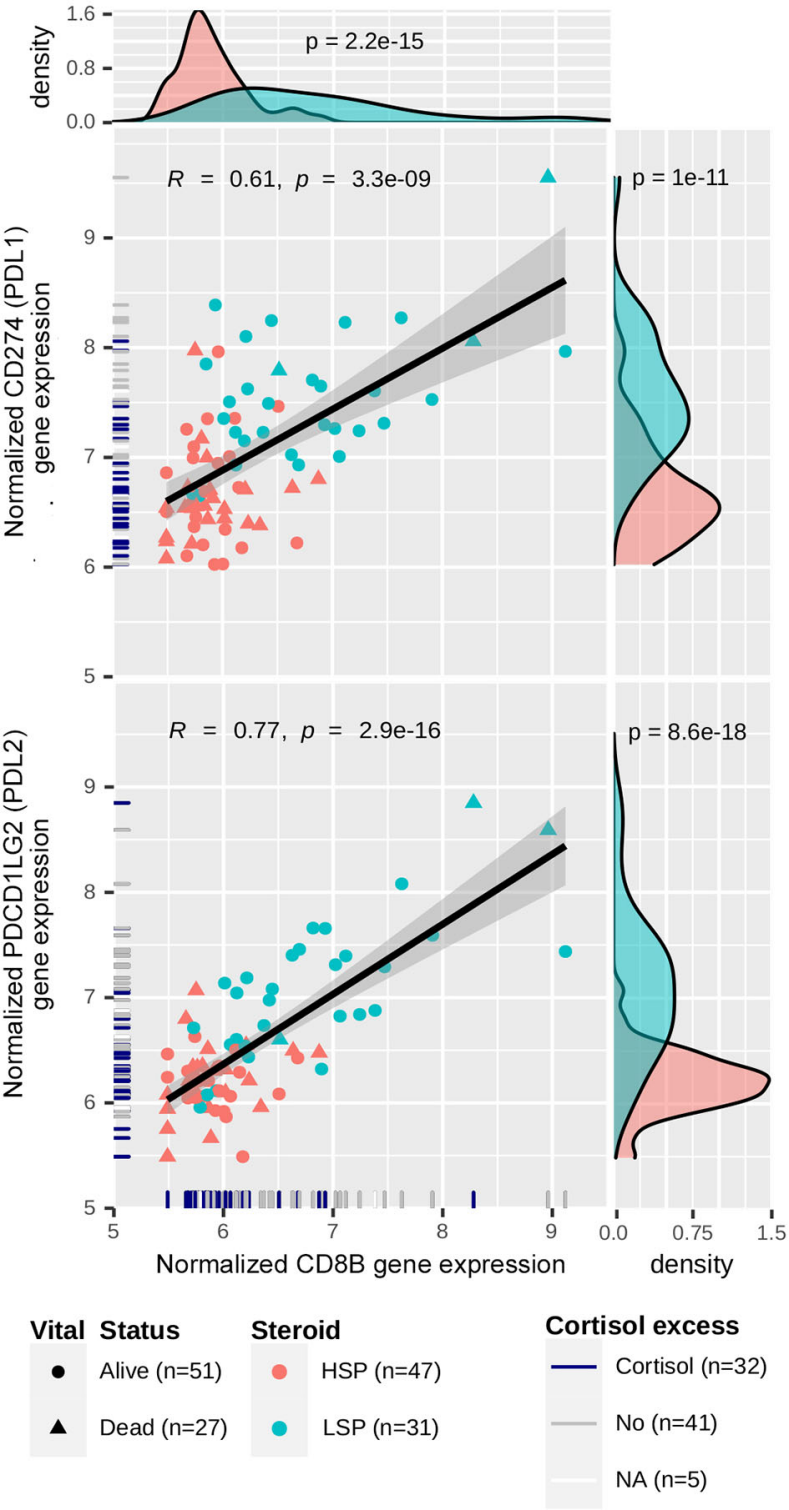
Correlation between *CD274/PDCD1LG2* and *CD8B* expression. Gene expression levels for *CD274, PDCD1LG2* and *CD8B* were normalized using a variance-stabilizing transformation from DESeq2 R package. Pearson’s correlation coefficients and p-values are shown in the scatter plots, as well as the regression line. The confidence interval was inferred using Fisher’s Z transform. Point colors are set according to the steroid phenotype: salmon for HSP and blue for LSP. Point shapes are set according to the patient’s vital status: triangles for dead and circles for alive. In the rug plot, navy blue indicates the presence of cortisol excess, gray indicates absence, and white indicates non-annotated patients regarding cortisol levels. In the density plot, p-values determined using Wald’s test for DE analysis between LSP and HSP is shown.

In the C3 network, which was mainly associated with patients with LSP (**Figure 7A**), antigen presentation molecules and some of their receptors were prominently present. It is noteworthy that *PDCD1LG2 (PD-L2)*, but not *CD274* (*PD-L1)*, appeared in good concordance with its receptor *PDCD1 (PD-1)*. Interestingly, a relative positive correlation was observed between *CD8B* and *CD274* expression, but it was more evident between *CD8B* and *PDCD1LG2* expression (**Figure 8**).

Finally, *CD276 (B7-H3)* and *FLT1* (*VEGFR1)* were present in abundance in both networks. *CD276* was in concordance with CD8+ T cells in C3 and in concordance with dendritic cells in C4, while *FLT1* was in concordance with *VEGFA* in C4 and in concordance with *VEGFB* in C3.

### Steroid Phenotype Was More Predictable of Outcome Than Immune Subtype Alone

**Figure 9:**
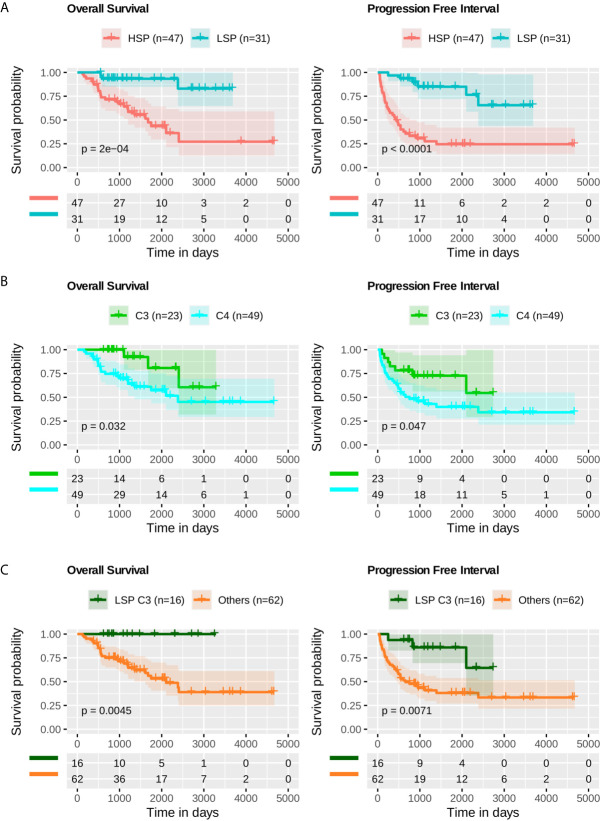
Survival analysis. Kaplan-Meier analysis for overall survival (OS) and progression free interval (PFI) of ACC profiles. The p-value was calculated using a log-rank test and the confidence interval is shown. Below each plot a survival table with the absolute number of participants over time. **(A)** OS and PFI for LSP (n = 31) versus HSP (n = 47). **(B)** OS and PFI for immune subtype C3 (n = 23) versus immune subtype C4 (n = 49). **(C)** OS and PFI for LSP and C3 patients (n = 16) versus other combinations (LSP C2, C4, C5, and C6, or HSP C1, C3, C4, and C5; total n = 62).

Based on Kaplan-Meier analysis, LSP presented better outcomes, both in OS (p = 2 × 10^-4^) and PFI (p-value < 1 × 10^–4^) (**Figure 9A**). When comparing the C3 and C4 immune subtypes, a slightly better prognosis was seen for C3 (p = 3.2 × 10^-2^ for OS and p = 4.7 × 10^-2^ for PFI) (**Figure 9B**). The separation between C3 and patients with LSP from the other configurations showed a significant difference in survival analysis (p = 4.5 × 10^-3^ for OS and p = 7.1 × 10^-3^ for PFI) (**Figure 9C**).

## Discussion

We present a complementary approach to the analyses of the pioneering studies by Zheng et al. (5) and Thorsson et al. (11) and revisit the potential biomarkers underlying the immunological processes in ACC, considering important contributions from previous studies (7–9, 12)⁠. The interplay between steroid production and the immune response in ACC has been a topic of recent studies (5, 12, 14, 18, 19)⁠. We have uncovered further interesting findings from the ACC datasets, deepened the investigation of immune pathways, and revealed possible druggable targets that may guide the selection criteria for ACC-targeted immunotherapy.

We demonstrated the immune system capabilities were dramatically better in patients with LSP (**Figures 1** and **2**), which is in agreement with studies describing the immune suppression caused by excess glucocorticoids (12)⁠. This higher immune activation seen in a subset of patients with ACC was in contrast with worst outcomes previously demonstrated for ACC in a pan-cancer analysis of solid tumors, which considered all patients with ACC without distinction of subtypes (5, 11). Our analyses, which considered steroid phenotype, improved our understanding of how the immune system reacts to ACC.

The immune cell composition in the TME is associated with important prognostic and therapeutic characteristics (14, 39)⁠. Our analysis of TME was based on scores inferred by previous studies (11, 39)⁠ and used two different methods, xCell and Cibersort, to detect immune cells according to bulk RNA-seq data. Some observed divergences (e.g., native CD4+ T cells) may be explained by limitation of the methods, such as background prediction or the spillover effect, as discussed by Sturm et al. (39)⁠. However, a higher immune cell infiltrate was observed in general for patients with LSP compared with that for patients with HSP (**Figures 5A, B**). Furthermore, both methods showed a significantly higher presence of cells associated with cytotoxic activity (**Figure 5B**), such as CD8+ T cells (p = 4.3 × 10^−8^ for xCell and p = 7 × 10^−8^ for Cibersort scores), T NK cells (p = 1.1 × 10^−5^ for xCell scores), and activated NK cells (p = 3.8 × 10^−6^ for Cibersort scores).

Notably, we observed an activation of immune system-related genes (**Figure 6A**), as well as expression of immune evasion-related genes in patients with LSP (**Figures 6A** and **8**). We also demonstrated a positive correlation between *CD8B* and *CD274 (PD-L1)* expression (R = 0.61, p = 1 × 10^−11^) and *CD8B* and *PDCD1LG2* (*PD-L2)* (R = 0.77, p = 8.6 × 10^−18^) (**Figure 8**). This may indicate that selective pressure induced enhanced immune evasion by tumor cells, which is in agreement with recent studies (51)⁠. PD-1/PD-L1-related and PD-1/PD-L2-related immune escape mechanisms are known to downregulate the CD8+ T-cell activation process. *CD274* is expressed by other immune cells in greater a proportion than that of *PDCD1LG2* expression (58)⁠, which may help explain why the dispersion seen in scatter plots was more evident for *CD274*. In addition, patients with HSP ACC overall presented lower levels of *CD8B* and *CD274/PDCD1LG2* expression compared to those of patients with LSP ACC. However, the positive correlation observed between *CD8B* and *CD274/PDCD1LG2* expression may imply that following glucocorticoid suppression, mechanisms of immune evasion may be induced in patients with HSP ACC. The correlation between CD8+TL and PD-L1 expression levels cannot be determined using immunohistochemistry (14, 59)⁠. However, findings from a previous study (59)⁠ and our current study suggest that PD-L2 plays a greater role than that of PD-L1 in ACC cells (**Figure 6A**). Nevertheless, this hypothesis has not been assessed in a clinical trial. Anti-PD-1 clinical trials conducted without inhibiting steroidogenesis (30–32)⁠ may have had modest results due to the immune suppression caused by hormone production in the TME. On the other hand, Le Tourneau et al. (33)⁠ evaluated the concomitant suppression of steroidogenesis with and without mitotane administration. However, only anti-PD-L1 was used in their study and they did not consider the possibility of PD-L2 being an important immune checkpoint in ACC.

In agreement with recent findings showing that 90% of ACC samples (n = 48) were positive for B7-H3 (*CD276*) in immunohistochemical assays (60)⁠, our analysis demonstrated that *CD276* overexpression was detected in both LSP and HSP. Remarkably, its expression was considered high in a pan-cancer comparison of most ACC patients (**Figure 7B**). Moreover, *CD276* overexpression has been implicated in resistance to anti-PD1 therapies (61–63)⁠. B7-H3 is a member of the B7 ligand family and is a recent and attractive target for antibody-based immunotherapy. B7-H3 is differentially overexpressed in malignant cells at high frequency (60% of 25,000 tumor samples), is detected at low levels in normal tissues, and probably has an inhibitory role in adaptive immunity, suppressing T-cell activation and proliferation (60–62)⁠. B7-H3 modulates T-cell responses, but unlike PD-1 and CTLA-4, may inhibit T-cell activation, possibly by interacting with tumor necrosis family receptors (64)⁠. However, other investigators believe the B7-H3 receptor remains unknown (61). Recent studies have shown that blocking B7-H3 results in a response of tumor resistance to treatments with anti-PD1/PD-L1 antibodies (61)⁠. Yonesaka et al. (63)⁠ demonstrated the dual blockade of B7-H3 and PD-L1 enhances the antitumor reaction compared to that of PD-L1 blockade alone, which may indicate a role of B7-H3 overexpression in immune evasion. These findings suggest that anti-B7-H3 combined with anti-PD-1/PD-L1 antibody therapy is a promising approach for B7-H3-expressing cancers, such as ACC. In addition to *CD276* (B7-H3), *FLT1* (VEGFR1) appeared in both C3 and C4 networks (**Figure 7A**) and was highly expressed in ACC compared with that in other cancers (**Figure 7B**). This may imply a role in angiogenesis, which has been described as an important pathway in ACC (65, 66)⁠. However, clinical trials to date using VEGFR inhibitors for ACC have demonstrated limited results (67, 68)⁠.

In contrast to *CD276* (B7-H3) and *FLT1* (VEGFR1), *BTLA*, interleukin 10 (*IL10*), IL10 receptor subunit alpha *(IL10RA)*, integrin beta chain-2 (*ITGB2*), *CD274* (*PD-L1*), and *PDCD1LG2* (*PD-L2*) are significantly downregulated in HSP (**Supplementary Table 1**, **Figures 6A** and **7B**). Similar to PD-1 and CTLA-4, BTLA is an immune checkpoint associated with suppression of the immune response and its blockade has been related to a reduction of ovarian carcinoma *via* the regulation of IL6 and IL10 (69)⁠. IL10 mediates immunosuppressive signals through its interaction with IL10 receptors, leading to inhibition of synthesis of proinflammatory cytokines (70)⁠. Integrins, such as ITGB2, play significant roles in cellular adhesion and cell surface signaling, and also participate in leukocyte adhesion and extravasation to inflame tissues, and mediate the formation of immunological synapses (71)⁠.

Curiously, *TNFSF9 (4-1BB-L)* was present at a surprisingly high abundance in ACC when compared to that of other TCGA tumor types, but not its receptor *TNFRSF9* (*4-1BB)*, which was present mainly on CD8+ T cells, (**Figure 7B**). In the C4 network, *TNFSF9* appeared in concordance with dendritic and NK cells (**Figure 7A**). TNFSF9 is commonly expressed by antigen-presenting cells and its interaction with its receptor TNFRSF9 tends to stimulate T cells and cytotoxic activity, demonstrating strong anti-tumor activity (72)⁠. Although the use of TNFSF9 analogues as an immunotherapy strategy has been considered (73)⁠, the relatively low level expression of its receptor TNFRSF9 on T cells may limit the efficacy of this type of treatment. Understanding the role of *TNFSF9* overexpression in ACC may be a subject of future studies.

Classification of ACC into subgroups based on methylation profiles, which was also proposed by Zheng et al. (5), appears to be robust as reviewed by Crona and Beuschlein (6). However, despite providing good clinical prognostic indications, this classification has not resulted in novel treatment possibilities for each of the groups (6, 7, 74)⁠. One strong point of our current study using the molecular classification of steroidal profiles was the possibility of separating the immune activation profile of tumor subtypes. This is in agreement with the suggestion by Fiorentini et al. (7)⁠ that patients who may benefit from immunotherapies should be selected based on some type of molecular classification. We believe that separation based on steroid profiles may provide useful information regarding this criterion. Furthermore, the LSP classification successfully grouped all patients with higher expression of immune modulator genes, in addition to higher leukocyte fractions. Remarkably, the immune activation distinction was not clear when using the classification of cases based solely on clinical parameters, that is, hypercortisolism versus non-hypercortisolism (data not shown). This was despite its good correlation with HSP (Fisher’s exact test, p = 2.55 × 10^−4^). In addition, the available HSP data are associated with downregulation of the immune system, higher tumor proliferation, and worst prognosis (75)⁠.

Genomic and epigenetic changes frequently occur in ACC, in both childhood (15, 76)⁠ and adulthood (5, 75, 77) cases. However, the TCGA ACC cohort is mainly limited to adults (median age, 46 years). The accumulation of these alterations may alter lymphocyte recruitment (11) and drive a higher proliferation profile, which would consequently result in a worse prognosis. It is currently not clear if the anti-tumor immune response is not strong enough to surpass tumor proliferation or if there is a surge in proliferation due to the immunosuppressed TME.

Interestingly, it was not possible to determine a clear correlation between leukocyte fraction and mutations in driver gene known to impact the immune response. This could have been due to the small number of patients with these mutations, as well as the fact that most of these patients were from the immunosuppressed HSP group. In analysis of the whole cohort (LSP + HSP patients) *CTNNB1* mutations were noted only in patients of the HSP group and they demonstrated a weak negative correlation with leukocyte fraction (p = 3.8 × 10^−2^ on Wilcoxon test). However, when analyzing only the HSP group, the presence of *CTNNB1* mutations did not demonstrate any significant impact on leukocyte fraction (p = 9.9 × 10^−1^ on Wilcoxon test), indicating the weak correlation observed was mainly due to the steroid phenotype. This indicates the steroid phenotype should be taken into account during immune response analysis of ACC. Failing to do so may generate confounding effects.

When comparing the inflammatory C3 and lymphocyte-depleted C4 immune signatures, we found a slightly better survival for C3 (p = 3.2 × 10^−2^ for OS and p = 4.7 × 10^−2^ for PFI; log-rank test) (**Figure 9B**). In the pan-cancer analysis performed by Thorsson and colleagues, C3 had a significantly better outcome than that of C4; however, the steroid phenotype provides more characteristics regarding tumor aggressiveness and outcome prognosis in ACC than that of the C3-C4 signatures. For example, there were no death events among the 16 patients with C3 LSP, whereas there were 3 deaths among the 7 patients with C3 HSP (42.9%). Furthermore, among the 12 patients with C4 LSP, only 1 died (8.33%) compared with 19 deaths in the group of 37 patients with C4 HSP (51,35%). When distinguishing C3 and C4 patients within the steroid phenotypes (density plots in **Figures 5B**, **6B**), differences were observed in LSP C3 versus C4, especially in the leukocyte fraction and lymphocyte infiltration scores; however, HSP C3 versus C4 does not in general present great distinction. Considering there were only seven patients with HSP C3, the steroid phenotype distinguished some immune features better than that of the C1–C6 subtype classification alone.

In addition to differences in the targeted immune checkpoints, a crucial difference affecting the success of immunotherapies for other tumors (22–24, 78)⁠ and the modest results of immunotherapy in ACC, such as less than 15% of patients with ACC benefiting from PD-1 or PD-L1 inhibition (30–33)⁠, seems to be the immunosuppressive and anti-inflammatory effects of intratumoral glucocorticoids (12)⁠. Among potential prognostic factors, CD3+ and CD8+ cytotoxic T lymphocytes were found in 84% of 146 patients with ACC and were associated with overall and recurrence-free survival (12)⁠. This parameter could be further improved by blocking steroidogenesis in combination with different immune checkpoint inhibitors. Patients with tumor progression and mitotane-resistance may still benefit from a reduction in the dose aiming for steroidogenesis blockade. One important feature of mitotane treatment is the ability to block steroid biosynthesis at lower daily doses and with less toxic side effects (approximately <10 µg/mL of plasma) (79)⁠. Future studies may investigate if this blockade is adequate to induce an efficient immune response.

Despite the reduced number of cases, we found evidence of considerable immune cell infiltrates within LSP and sought to characterize the interactions and networks in order to identify transcriptomic biomarkers associated with significant immune activation. Our analysis also exploited target immune modulators that may regulate potential pathways associated with the anti-tumor response. Our analysis framework had limitations as it relied on a small number of accessible profiles and a simple binary change (LSP and HSP) where HSP combined different steroid subtypes that could not be identified using only the clinical manifestations of hypercortisolism. It is important to note that the activation of steroid biosynthesis pathways seen in HSP was based on a transcriptome profile, which may imply a possible hormone excess in the TME had not been related to clinical manifestations. Our experimental design using a probabilistic approach to identify potential targets for immunotherapy was restricted to *in silico* findings. Despite the rationale, this may not be translatable to *in vivo* assays. Further work is needed to fully understand the possibility of changing the outcome of ACC immunotherapy, which may start with the selection of patients with LSP ACC and the inhibition of steroidogenesis in patients with HSP ACC. Despite significant progress in identifying immunogenic biomarkers in ACC through evaluation of the TCGA cohort (5), there are still many limitations and puzzles to be solved. Additional efforts beyond RNAseq analyses are needed to validate functional studies to define the key pathways in a larger number of patients with LSP ACC. Considering we are still in the early stage of understanding the B7-H3 network, it is important to define the actual B7-H3 receptor in order to fully understand this pathway in ACC.

## Conclusions

An important frontier of ACC therapeutics to improve the anti-tumor activity of the immune system starts explicitly by modeling steroidogenic differences. The analytical approach we describe herein requires further studies to ascertain the possible druggable role of B7-H3 in relation to PD1-PDL1/PDL2 in ACC. Our present approach was unable to predict improved outcomes of immunotherapy; however, it points to possible selection criteria and potential targets. Future work should confirm these findings using a larger cohort, in addition to including pre-clinical or clinical trials.

## Code Availability and Reproducibility 

All scripts, datasets, software and algorithms used to generate results, figures, and tables for this study are available on the GitHub repository (https://github.com/sysbiolab/Sup_Material_Muzzi2021) and **Supplementary Tables 3** and **4**.

## Data Availability Statement

The original contributions presented in the study are included in the article/**Supplementary Material**. Further inquiries can be directed to the corresponding author. 

## Ethics Statement

The studies involving human participants were reviewed and approved by Pequeno Príncipe Ethics Committee. Written informed consent from the participants’ legal guardian/next of kin was not required to participate in this study in accordance with the national legislation and the institutional requirements.

## Author Contributions

All authors made substantial contributions to the conception of the work. JCDM was responsible for data acquisition and analysis. MAAC contributed to the acquisition of data and method analyses. All authors contributed to data interpretation. JCDM and BF wrote the first draft of the manuscript. JMM and MAC helped in code review and creating the public repository. All authors contributed to the article and approved the submitted version.

## Funding

JCDM, JMM, and MAC receive scholarships from the BIG DATA innovation program from the Associação Hospitalar de Proteção à Infância Raul Carneiro-AHPIRAC (2020). MAAC and JM are funded by the Conselho Nacional de Desenvolvimento Científico e Tecnológico (CNPq).

## Conflict of Interest

The authors declare the current research was conducted in the absence of any commercial or financial relationships that could be construed as a potential conflict of interest.

## References

[1] Allolio B Fassnacht M. Adrenocortical Carcinoma: Clinical Update. J Clin Endocrinol Metab (2006) 91:2027–37. 10.1210/jc.2005-2639 16551738

[2] Libè R Fratticci A Bertherat J. Adrenocortical Cancer: Pathophysiology and Clinical Management. Endocr Relat Cancer (2007) 14:13–28. 10.1677/erc.1.01130 17395972

[3] Fassnacht M Kroiss M Allolio B. Update in Adrenocortical Carcinoma. J Clin Endocrinol Metab (2013) 98:4551–64. 10.1210/jc.2013-3020 24081734

[4] Berruti A Fassnacht M Haak H Else T Baudin E Sperone P. Prognostic Role of Overt Hypercortisolism in Completely Operated Patients With Adrenocortical Cancer. Eur Urol (2014) 65:832–38. 10.1016/j.eururo.2013.11.006 24268504

[5] Zheng S Cherniack AD Dewal N Moffitt RA Danilova L Murray BA. Comprehensive Pan-Genomic Characterization of Adrenocortical Carcinoma. Cancer Cell (2016) 29:723–36. 10.1016/j.ccell.2016.04.002 PMC4864952 27165744

[6] Crona J Beuschlein F. Adrenocortical Carcinoma — Towards Genomics Guided Clinical Care. Nat Rev Endocrinol (2019) 15:548–60. 10.1038/s41574-019-0221-7 31147626

[7] Fiorentini C Grisanti S Cosentini D Abate A Rossini E Berruti A. Molecular Drivers of Potential Immunotherapy Failure in Adrenocortical Carcinoma. J Oncol (2019) 2019:6072863. 10.1155/2019/6072863 31057613 PMC6463568

[8] de Reyniès A Assié G Rickman DS Tissier F Groussin L René-Corail F. Gene Expression Profiling Reveals a New Classification of Adrenocortical Tumors and Identifies Molecular Predictors of Malignancy and Survival. J Clin Oncol (2009) 27:1108–15. 10.1200/JCO.2008.18.5678 19139432

[9] Giordano TJ Kuick R Else T Gauger PG Vinco M Bauersfeld J. Molecular Classification and Prognostication of Adrenocortical Tumors by Transcriptome Profiling. Clin Cancer Res (2009) 15:668–76. 10.1158/1078-0432.CCR-08-1067 PMC2629378 19147773

[10] Li B Severson E Pignon J-CC Zhao H Li T Novak J. Comprehensive Analyses of Tumor Immunity: Implications for Cancer Immunotherapy. Genome Biol (2016) 17:174. 10.1186/s13059-016-1028-7 27549193 PMC4993001

[11] Thorsson V Gibbs DL Brown SD Wolf D Bortone DS Ou Yang TH. The Immune Landscape of Cancer. Immunity (2018) 48:812–30.e14. 10.1016/j.immuni.2018.03.023 29628290 PMC5982584

[12] Landwehr L-S Altieri B Schreiner J Sbiera I Weigand I Kroiss M. Interplay Between Glucocorticoids and Tumor-Infiltrating Lymphocytes on the Prognosis of Adrenocortical Carcinoma. J Immunother Cancer (2020) 8:e000469. 10.1136/jitc-2019-000469 32474412 PMC7264832

[13] Michalkiewicz E Sandrini R Figueiredo B Miranda ECMM Caran E Oliveira-Filho AG. Clinical and Outcome Characteristics of Children With Adrenocortical Tumors: A Report From the International Pediatric Adrenocortical Tumor Registry. J Clin Oncol (2004) 22:838–45. 10.1200/JCO.2004.08.085 14990639

[14] Parise IZS Parise GA Noronha L Surakhy M Woiski TD Silva DB. The Prognostic Role of CD8+ T Lymphocytes in Childhood Adrenocortical Carcinomas Compared to Ki-67, Pd-1, PD-L1, and the Weiss Score. Cancers (Basel) (2019) 11:1730. 10.3390/cancers11111730 PMC6896110 31694270

[15] Pinto EM Chen X Easton J Finkelstein D Liu Z Pounds S. Genomic Landscape of Paediatric Adrenocortical Tumours. Nat Commun (2015) 6:6302. 10.1038/ncomms7302 25743702 PMC4352712

[16] Finn OJ. Cancer Immunology. N Engl J Med (2008) 358:2704–15. 10.1056/NEJMra072739 18565863

[17] Custódio G Komechen H Figueiredo FRO Fachin ND Pianovski MAD Figueiredo BC. Molecular Epidemiology of Adrenocortical Tumors in Southern Brazil. Mol Cell Endocrinol (2012) 351:44–51. 10.1016/j.mce.2011.10.019 22056871

[18] Vanbrabant T Fassnacht M Assie G Dekkers OM. Influence of Hormonal Functional Status on Survival in Adrenocortical Carcinoma: Systematic Review and Meta-Analysis. Eur J Endocrinol (2018) 179:429–36. 10.1530/EJE-18-0450 30325179

[19] Lalli E Figueiredo BC. Pediatric Adrenocortical Tumors: What They Can Tell Us on Adrenal Development and Comparison With Adult Adrenal Tumors. Front Endocrinol (Lausanne) (2015) 6:23. 10.3389/fendo.2015.00023 25741319 PMC4332354

[20] Prager I Liesche C van Ooijen H Urlaub D Verron Q Sandström N. NK Cells Switch From Granzyme B to Death Receptor–Mediated Cytotoxicity During Serial Killing. J Exp Med (2019) 216:2113–27. 10.1084/jem.20181454 PMC6719417 31270246

[21] Cursons J Souza-Fonseca-Guimaraes F Foroutan M Anderson A Hollande F Hediyeh-Zadeh S. A Gene Signature Predicting Natural Killer Cell Infiltration and Improved Survival in Melanoma Patients. Cancer Immunol Res (2019) 7:1162–74. 10.1158/2326-6066.CIR-18-0500 31088844

[22] Drake CG Lipson EJ Brahmer JR. Breathing New Life Into Immunotherapy: Review of Melanoma, Lung and Kidney Cancer. Nat Rev Clin Oncol (2014) 11:24–37. 10.1038/nrclinonc.2013.208 24247168 PMC4086654

[23] Rodriguez D Goulart C Pagliarone AC Silva EP Cunegundes PS Nascimento IP. *In Vitro* Evidence of Human Immune Responsiveness Shows the Improved Potential of a Recombinant Bcg Strain for Bladder Cancer Treatment. Front Immunol (2019) 10:1460. 10.3389/fimmu.2019.01460 31297119 PMC6607967

[24] Pettenati C Ingersoll MA. Mechanisms of BCG Immunotherapy and its Outlook for Bladder Cancer. Nat Rev Urol (2018) 15:615–25. 10.1038/s41585-018-0055-4 29991725

[25] Hong H Wang Q Li J Liu H Meng X Zhang H. Aging, Cancer and Immunity. J Cancer (2019) 10:3021–7. 10.7150/jca.30723 PMC6590045 31281479

[26] Wasserman JD Zambetti GP Malkin D. Towards an Understanding of the Role of p53 in Adrenocortical Carcinogenesis. Mol Cell Endocrinol (2012) 351:101–10. 10.1016/j.mce.2011.09.010 PMC3288384 21930187

[27] Bindea G Mlecnik B Tosolini M Kirilovsky A Waldner M Obenauf AC. Spatiotemporal Dynamics of Intratumoral Immune Cells Reveal the Immune Landscape in Human Cancer. Immunity (2013) 39:782–95. 10.1016/j.immuni.2013.10.003 24138885

[28] Rieder SA Wang J White N Qadri A Menard C Stephens G. B7-H7 (HHLA2) Inhibits T-cell Activation and Proliferation in the Presence of TCR and CD28 Signaling. Cell Mol Immunol (2020). 10.1038/s41423-020-0361-7 PMC8166953 32005952

[29] Greenwald RJ Freeman GJ Sharpe AH. The B7 Family Revisited. Annu Rev Immunol (2005) 23:515–48. 10.1146/annurev.immunol.23.021704.115611 15771580

[30] Carneiro BA Konda B Costa RB Costa RLB Sagar V Gursel DB. Nivolumab in Metastatic Adrenocortical Carcinoma: Results of a Phase 2 Trial. J Clin Endocrinol Metab (2019) 104:6193–200. 10.1210/jc.2019-00600 31276163

[31] Raj N Zheng Y Kelly V Katz SS Chou J Do RKG. Pd-1 Blockade in Advanced Adrenocortical Carcinoma. J Clin Oncol (2020) 38:71–80. 10.1200/JCO.19.01586 31644329 PMC7351334

[32] Habra MA Stephen B Campbell M Hess K Tapia C Xu M. Phase II Clinical Trial of Pembrolizumab Efficacy and Safety in Advanced Adrenocortical Carcinoma. J Immunother Cancer (2019) 7:253. 10.1186/s40425-019-0722-x 31533818 PMC6751592

[33] Le Tourneau C Hoimes C Zarwan C Wong DJ Bauer S Claus R. Avelumab in Patients With Previously Treated Metastatic Adrenocortical Carcinoma: Phase 1b Results From the JAVELIN Solid Tumor Trial. J Immunother Cancer (2018) 6:111. 10.1186/s40425-018-0424-9 30348224 PMC6198369

[34] Cosentini D Grisanti S Volta AD Laganà M Fiorentini C Perotti P. Immunotherapy Failure in Adrenocortical Cancer: Where Next? Endocr Connect (2018) 7:E5–8. 10.1530/EC-18-0398 PMC6280582 30400026

[35] Colaprico A Silva TC Olsen C Garofano L Cava C Garolini D. Tcgabiolinks: An R/Bioconductor Package for Integrative Analysis of TCGA Data. Nucleic Acids Res (2016) 44:e71. 10.1093/nar/gkv1507 26704973 PMC4856967

[36] Silva TC Colaprico A Olsen C D’Angelo F Bontempi G Ceccarelli M. Tcga Workflow: Analyze Cancer Genomics and Epigenomics Data Using Bioconductor Packages. F1000Research (2016) 5:1542. 10.12688/f1000research.8923.2 28232861 PMC5302158

[37] Mounir M Lucchetta M Silva TC Olsen C Bontempi G Chen X. New Functionalities in the TCGAbiolinks Package for the Study and Integration of Cancer Data From GDC and Gtex. PloS Comput Biol (2019) 15:e1006701. 10.1371/journal.pcbi.1006701 30835723 PMC6420023

[38] Aran D Hu Z Butte AJ. xCell: Digitally Portraying the Tissue Cellular Heterogeneity Landscape. Genome Biol (2017) 18:220. 10.1186/s13059-017-1349-1 29141660 PMC5688663

[39] Sturm G Finotello F Petitprez F Zhang JD Baumbach J Fridman WH. Comprehensive Evaluation of Transcriptome-Based Cell-Type Quantification Methods for Immuno-Oncology. Bioinformatics (2019) 35:i436–45. 10.1093/bioinformatics/btz363 PMC6612828 31510660

[40] Newman AM Liu CL Green MR Gentles AJ Feng W Xu Y. Robust Enumeration of Cell Subsets From Tissue Expression Profiles. Nat Methods (2015) 12:453–7. 10.1038/nmeth.3337 PMC4739640 25822800

[41] Gu Z Eils R Schlesner M. Complex Heatmaps Reveal Patterns and Correlations in Multidimensional Genomic Data. Bioinformatics (2016) 32:2847–9. 10.1093/bioinformatics/btw313 27207943

[42] Morgan M Obenchain V Hester J Pagès H. Summarizedexperiment: SummarizedExperiment Container. In: R package version 1.20.0 (2020). 10.18129/B9.bioc.SummarizedExperiment

[43] Morgan M Shepherd L. Annotationhub: Client to Access AnnotationHub Resources. In: R package, version 2.22.0 (2020). 10.18129/B9.bioc.AnnotationHub

[44] Love MI Huber W Anders S. Moderated Estimation of Fold Change and Dispersion for RNA-seq Data With Deseq2. Genome Biol (2014) 15:550. 10.1186/s13059-014-0550-8 25516281 PMC4302049

[45] Yamaguchi KD Ruderman DL Croze E Wagner TC Velichko S Reder AT. Ifn-β-Regulated Genes Show Abnormal Expression in Therapy-Naïve Relapsing–Remitting MS Mononuclear Cells: Gene Expression Analysis Employing All Reported Protein–Protein Interactions. J Neuroimmunol (2008) 195:116–20. 10.1016/j.jneuroim.2007.12.007 18279974

[46] Weiner J 3rd Domaszewska T. Tmod: An R Package for General and Multivariate Enrichment Analysis. PeerJ (2016) 4:1–9. 10.7287/peerj.preprints.2420

[47] Zyla J Marczyk M Domaszewska T Kaufmann SHE Polanska J Weiner J. Gene Set Enrichment for Reproducible Science: Comparison of CERNO and Eight Other Algorithms. Bioinformatics (2019) 35:5146–54. 10.1093/bioinformatics/btz447 PMC6954644 31165139

[48] Subramanian A Tamayo P Mootha VK Mukherjee S Ebert BL Gillette MA. Gene Set Enrichment Analysis: A Knowledge-Based Approach for Interpreting Genome-Wide Expression Profiles. Proc Natl Acad Sci (2005) 102:15545–50. 10.1073/pnas.0506580102 PMC1239896 16199517

[49] Liberzon A Birger C Thorvaldsdóttir H Ghandi M Mesirov JP Tamayo P. The Molecular Signatures Database Hallmark Gene Set Collection. Cell Syst (2015) 1:417–25. 10.1016/j.cels.2015.12.004 PMC4707969 26771021

[50] Li S Rouphael N Duraisingham S Romero-Steiner S Presnell S Davis C. Molecular Signatures of Antibody Responses Derived From a Systems Biology Study of Five Human Vaccines. Nat Immunol (2014) 15:195–204. 10.1038/ni.2789 24336226 PMC3946932

[51] Wang H Najibi AJ Sobral MC Seo BR Lee JY Wu D. Biomaterial-Based Scaffold for in Situ Chemo-Immunotherapy to Treat Poorly Immunogenic Tumors. Nat Commun (2020) 11:5696. 10.1038/s41467-020-19540-z 33173046 PMC7655953

[52] Ramilowski JA Goldberg T Harshbarger J Kloppmann E Lizio M Satagopam VP. A Draft Network of Ligand–Receptor-Mediated Multicellular Signalling in Human. Nat Commun (2015) 6:7866. 10.1038/ncomms8866 26198319 PMC4525178

[53] Castro MA Wang X Fletcher MN Meyer KB Markowetz F. Reder: R/Bioconductor Package for Representing Modular Structures, Nested Networks and Multiple Levels of Hierarchical Associations. Genome Biol (2012) 13:R29. 10.1186/gb-2012-13-4-r29 22531049 PMC3446303

[54] Hoadley KA Yau C Hinoue T Wolf DM Lazar AJ Drill E. Cell-of-Origin Patterns Dominate the Molecular Classification of 10,000 Tumors From 33 Types of Cancer. Cell (2018) 173:291–304. 10.1016/j.cell.2018.03.022 29625048 PMC5957518

[55] Therneau T. A Package for Survival Analysis in R. In: R package version 3.2-7 (2020). Available at: https://cran.r-project.org/package=survival.

[56] Therneau TM Grambsch PM. Modeling Survival Data: Extending the Cox Model. New York, NY: Springer New York (2000). 10.1007/978-1-4757-3294-8

[57] Storey J Bass A Dabney A Robinson D. Qvalue: Q-value Estimation for False Discovery Rate Control R Package Version 2.22.0. (2020). 10.18129/B9.bioc.qvalue.

[58] Monaco G Lee B Xu W Mustafah S Hwang YY Carré C. Rna-Seq Signatures Normalized by Mrna Abundance Allow Absolute Deconvolution of Human Immune Cell Types. Cell Rep (2019) 26:1627–40.e7. 10.1016/j.celrep.2019.01.041 30726743 PMC6367568

[59] Tierney JF Vogle A Poirier J Min IM Finnerty B Zarnegar R. Expression of Programmed Death Ligand 1 and 2 in Adrenocortical Cancer Tissues: An Exploratory Study. Surgery (2019) 165:196–201. 10.1016/j.surg.2018.04.086 30413322

[60] Liang J Liu Z Pei T Xiao Y Zhou L Tang Y. Clinicopathological and Prognostic Characteristics of CD276 (B7-H3) Expression in Adrenocortical Carcinoma. Dis Markers (2020) 2020:1–10. 10.1155/2020/5354825 PMC6977319 31998416

[61] Kontos F Michelakos T Kurokawa T Sadagopan A Schwab JH Ferrone CR. B7-H3: An Attractive Target for Antibody-based Immunotherapy. Clin Cancer Res (2021) 27:1227–35. 10.1158/1078-0432.CCR-20-2584 PMC7925343 33051306

[62] Flem-Karlsen K Fodstad Ø Tan M Nunes-Xavier CE. B7-H3 in Cancer – Beyond Immune Regulation. Trends Cancer (2018) 4:401–4. 10.1016/j.trecan.2018.03.010 29860983

[63] Yonesaka K Haratani K Takamura S Sakai H Kato R Takegawa N. B7-H3 Negatively Modulates CTL-Mediated Cancer Immunity. Clin Cancer Res (2018) 24:2653–64. 10.1158/1078-0432.CCR-17-2852 29530936

[64] Loos M Hedderich DM Friess H Kleeff J. B7-H3 and Its Role in Antitumor Immunity. Clin Dev Immunol (2010) 2010:1–7. 10.1155/2010/683875 PMC2993024 21127709

[65] Xu Y Zhu Y Shen Z Sheng J He H Ma G. Significance of Heparanase-1 and Vascular Endothelial Growth Factor in Adrenocortical Carcinoma Angiogenesis: Potential for Therapy. Endocrine (2011) 40:445–51. 10.1007/s12020-011-9502-1 21706269

[66] Pereira SS Costa MM Guerreiro SG Monteiro MP Pignatelli D. Angiogenesis and Lymphangiogenesis in the Adrenocortical Tumors. Pathol Oncol Res (2018) 24:689–93. 10.1007/s12253-017-0259-6 28695321

[67] O’Sullivan C Edgerly M Velarde M Wilkerson J Venkatesan AM Pittaluga S. The VEGF Inhibitor Axitinib has Limited Effectiveness as a Therapy for Adrenocortical Cancer. J Clin Endocrinol Metab (2014) 99:1291–7. 10.1210/jc.2013-2298 PMC3973787 24423320

[68] Berruti A Sperone P Ferrero A Germano A Ardito A Priola AM. Phase II Study of Weekly Paclitaxel and Sorafenib as Second/Third-Line Therapy in Patients With Adrenocortical Carcinoma. Eur J Endocrinol (2012) 166:451–8. 10.1530/EJE-11-0918 22189997

[69] Chen Y-L Lin H-W Chien C-L Lai Y-L Sun W-Z Chen C-A. BTLA Blockade Enhances Cancer Therapy by Inhibiting IL-6/IL-10-induced Cd19high B Lymphocytes. J Immunother Cancer (2019) 7:313. 10.1186/s40425-019-0744-4 31753019 PMC6868712

[70] Pestka S Krause CD Sarkar D Walter MR Shi Y Fisher PB. Interleukin-10 and Related Cytokines and Receptors. Annu Rev Immunol (2004) 22:929–79. 10.1146/annurev.immunol.22.012703.104622 15032600

[71] Rojas K Baliu-Piqué M Manzano A Saiz-Ladera C García-Barberán V Cimas FJ. In Silico Transcriptomic Mapping of Integrins and Immune Activation in Basal-like and HER2+ Breast Cancer. Cell Oncol (2021). 10.1007/s13402-020-00583-9 PMC12980691 33469836

[72] Vinay DS Kwon BS. 4-1BB Signaling Beyond T Cells. Cell Mol Immunol (2011) 8:281–4. 10.1038/cmi.2010.82 PMC4002439 21217771

[73] Chester C Sanmamed MF Wang J Melero I. Immunotherapy Targeting 4-1BB: Mechanistic Rationale, Clinical Results, and Future Strategies. Blood (2018) 131:49–57. 10.1182/blood-2017-06-741041 29118009

[74] Jasim S Habra MA. Management of Adrenocortical Carcinoma. Curr Oncol Rep (2019) 21:20. 10.1007/s11912-019-0773-7 30798468

[75] Assié G Letouzé E Fassnacht M Jouinot A Luscap W Barreau O. Integrated Genomic Characterization of Adrenocortical Carcinoma. Nat Genet (2014) 46:607–12. 10.1038/ng.2953 24747642

[76] Figueiredo BC Stratakis CA Sandrini R DeLacerda L Pianovsky MAD Giatzakis C. Comparative Genomic Hybridization Analysis of Adrenocortical Tumors of Childhood 1. J Clin Endocrinol Metab (1999) 84:1116–21. 10.1210/jcem.84.3.5526 10084604

[77] Kjellman M Kallioniemi OP Karhu R Höög A Farnebo LO Auer G. Genetic Aberrations in Adrenocortical Tumors Detected Using Comparative Genomic Hybridization Correlate With Tumor Size and Malignancy. Cancer Res (1996) 56:4219–23. 8797595

[78] Hodi FS O’Day SJ McDermott DF Weber RW Sosman JA Haanen JB. Improved Survival With Ipilimumab in Patients With Metastatic Melanoma. N Engl J Med (2010) 363:711723. 10.1056/NEJMoa1003466 PMC3549297 20525992

[79] Zancanella P Pianovski MAD Oliveira BH Ferman S Piovezan GC Lichtvan LL. Mitotane Associated With Cisplatin, Etoposide, and Doxorubicin in Advanced Childhood Adrenocortical Carcinoma. J Pediatr Hematol Oncol (2006) 28:513–24. 10.1097/01.mph.0000212965.52759.1c 16912591

